# Glandular radiation dose in tomosynthesis of the breast using tungsten targets

**DOI:** 10.1120/jacmp.v9i4.2887

**Published:** 2008-10-24

**Authors:** Ioannis Sechopoulosa, Carl J. D'Orsi

**Affiliations:** ^1^ Department of Radiology and Winship Cancer Institute Emory University School of Medicine Atlanta Georgia U.S.A.

**Keywords:** tomosynthesis, dosimetry, Monte Carlo, breast, mammography

## Abstract

With the advent of new detector technology, digital tomosynthesis imaging of the breast has, in the past few years, become a technique intensely investigated as a replacement for planar mammography. As with all other x‐ray–based imaging methods, radiation dose is of utmost concern in the development of this new imaging technology. For virtually all development and optimization studies, knowledge of the radiation dose involved in an imaging protocol is necessary. A previous study characterized the normalized glandular dose in tomosynthesis imaging and its variation with various breast and imaging system parameters. This characterization was performed with x‐ray spectra generated by molybdenum and rhodium targets. In the recent past, many preliminary patient studies of tomosynthesis imaging have been reported in which the x‐ray spectra were generated with x‐ray tubes with tungsten targets. The differences in x‐ray distribution among spectra from these target materials make the computation of new normalized glandular dose values for tungsten target spectra necessary. In this study we used previously obtained monochromatic normalized glandular dose results to obtain spectral results for twelve different tungsten target x‐ray spectra. For each imaging condition, two separate values were computed: the normalized glandular dose for the zero degree projection angle ($D_{g}N_{0}$), and the ratio of the glandular dose for non‐zero projection angles to the glandular dose for the zero degree projection (the relative glandular dose, RGD(α)). It was found that $D_{g}N_{0}$ is higher for tungsten target x‐ray spectra when compared with $D_{g}N_{0}$ values for molybdenum and rhodium target spectra of both equivalent tube voltage and first half value layer. Therefore, the $D_{g}N_{0}$ for the twelve tungsten target x‐ray spectra and different breast compositions and compressed breast thicknesses simulated are reported. The RGD(α) values for the tungsten spectra vary with the parameters studied in a similar manner to that found for the molybdenum and rhodium target spectra. The surface fit equations and the fit coefficients for RGD(α) included in the previous study were also found to be appropriate for the tungsten spectra.

PACS numbers: 87.57.uq

## I. INTRODUCTION

Digital tomosynthesis imaging of the breast^(^ ^1^ ^–^ ^3^ ^)^ is being intensely investigated as an alternative to conventional (planar) mammography. Several patient studies have already been reported, all with promising results.^(^ ^4^ ^–^ ^9^ ^)^ Tomosynthesis' ability to achieve enough vertical resolution to separate breast tissue that would appear superimposed in planar mammography seems to result in both lower recall rates^(^ ^6^ ^,^ ^7^ ^)^ and increased sensitivity.^(9)^

Given the x‐ray based nature of tomosynthesis imaging, and the target population of this imaging technique, namely screening of the general population and diagnostic workup, an accurate and comprehensive understanding of the radiation dosimetry characteristics of tomosynthesis imaging of the breast is of utmost importance. Previously, a computational dosimetry study was published to not only estimate the normalized glandular dose to the breast during tomosynthesis acquisition, but also to characterize its variation for varying breast sizes and composition and x‐ray spectra.^(10)^ That study reported the zero degree projections' (equivalent to planar mammography images) dosimetry characteristics separately from the results at non‐zero angles. The latter were reported using the relative glandular dose coefficient (RGD(α)), defined as the ratio of the glandular dose for the projection at angle a to the glandular dose for the zero degree projection under equal tube voltage and current settings. That study included results for both the craniocaudal (CC) and the medio‐lateral oblique (MLO) views, giving sufficient information to estimate the glandular dose to the breast from a complete two‐view study, be it a planar mammography or tomosynthesis examination.

Recently, several of the patient and simulation studies reporting the initial results of tomosynthesis imaging of the breast have been performed with x‐ray systems that use tungsten as the target material.^(^ ^4^ ^,^ ^11^ ^–^ ^14^ ^)^ X‐ray spectra from tungsten targets are very different from molybdenum or rhodium target spectra, and their dosimetric characteristics can be expected to reflect this difference in x‐ray energy distribution. The above mentioned tomosynthesis dosimetry study only included molybdenum and rhodium target x‐ray spectra, and therefore those results are not applicable to tomosynthesis examinations performed with x‐ray tubes with tungsten targets. Thus, the purpose of our study was to compute the normalized glandular dose from tomosynthesis imaging of the breast when the x‐ray tube used for acquisition of the projections has a tungsten target, and to study its variations with different breast characteristics and imaging parameters.

## II. MATERIALS AND METHODS

To obtain the spectral normalized glandular dose resulting from acquisitions performed using tungsten target x‐ray tubes, the monochromatic normalized glandular dose values obtained by Monte Carlo simulation for the previous study^(10)^ were re‐combined. In that study, a C++ program based on the Geant4 toolkit^(^ ^15^ ^,^ ^16^ ^)^ for Monte Carlo simulations was used to obtain monochromatic normalized glandular dose ($D_{g}$N(E)) estimates for x‐ray energies ranging from E=5.5 keV to 35.5 keV in 1 keV steps. For each of these energies, $D_{g}$N(E) values were obtained for breasts simulated to be in the CC and MLO views and with varying tomosynthesis projection angles ($\alpha =0^{0}$ to $\pm 30^{0}$ in 3° steps), breast composition (G=1%, 25%, 50%, 75% and 100% glandular fraction), breast chest wall to nipple distance (CND=7, 10, 13, 16 and 19 cm (MLO) and CND=6.2, 9.0, 11.6, 14.4 and 17.0 cm (CC)), and compressed breast thickness (T=2 cm to 8 cm in 1 cm steps). The computation of $D_{g}$N(E) in the Monte Carlo simulation was performed using the method described by Boone^(17)^ and Wilkinson and Heggie.^(18)^ These monochromatic results were combined to obtain spectral $D_{g}$N values for several different x‐ray spectra generated by molybdenum and rhodium targets using the method described by Thacker and Glick^(19)^ and the x‐ray spectra models published by Boone et al.^(20)^ For our study, the same monochromatic $D_{g}$N(E) were combined using the same methodology with several x‐ray spectra with tungsten targets, using the models published by Boone et al.^(20)^ The x‐ray spectra used, along with their resulting first half value layers are listed in Table 1. The filter materials and thicknesses chosen reflect those that are being used in some of the current tomosynthesis prototypes, namely 0.5 mm aluminum^(^ ^4^ ^,^ ^11^ ^)^ and 50 μm rhodium.^(^ ^21^ ^,^ ^22^ ^)^ The spectral model used, in combination with the mass attenuation coefficients of the filter materials obtained from Berger et al,^(23)^ resulted in first half value layers and/or mean energy values very similar to those reported for some prototype tomosynthesis machines.^(^ ^22^ ^,^ ^24^ ^,^ ^25^ ^)^

**Table 1:** First half‐value layer values (HVL) of the tungsten target x‐ray spectra used in this study.

*Filter*	*Tube Potential (kVp)*	*Computed HVL (mm Al)*	*HVL under Compression Plate (mm Al)*
Al	25	0.295	0.346
Al	27	0.322	0.382
Al	29	0.349	0.415
Al	31	0.376	0.450
Al	33	0.402	0.483
Al	35	0.429	0.518
Rh	25	0.447	0.475
Rh	27	0.473	0.502
Rh	29	0.492	0.522
Rh	31	0.510	0.540
Rh	33	0.525	0.557
Rh	35	0.542	0.575

As in the previous study,^(10)^ the $D_{g}$N for the zero degree projection angle ($D_{g}N_{0}$) was analyzed separately from that for the non‐zero projection angles. This separation was due to two reasons; (i) $D_{g}N_{0}$ can be used alone to compute glandular dose estimates for planar mammography imaging, and (ii) $D_{g}N_{0}$ is a function of all the parameters studied (breast size and composition, x‐ray spectrum and view), while, by using the RGD(α), the non‐zero projection angle dose estimates can be reported independently of x‐ray spectrum and breast composition.

The variation of the relative glandular dose, RGD(α), with the different parameters studied was analyzed by computing the mean and maximum of the coefficients of variation (COV=100σ/μ) and of the deviations from the mean of the RGD(α) values when each of the parameters is varied while maintaining all other parameters constant.

In the previous study, the following surface fit equations were provided for computation of the RGD(α) for any sized breast in either MLO or CC views:^(10)^

(1) $$RGD_{MLO}=\frac{a+ca+e\delta +g\alpha^{2}+i\delta^{2}+k\alpha \delta }{1+b\alpha +d\delta +f\alpha^{2}+h\delta^{2}+j\alpha \delta }$$

(2) $$RGD_{CC}=a+b\alpha +c\delta +d\alpha^{2}+e\delta^{2}+f\delta \alpha +g\alpha^{3}+h\delta^{3}+i\alpha \delta^{2}+j\alpha^{2}\delta$$

In these equations, a denotes the tomosynthesis angle (in degrees), and δ denotes the chest wall to nipple distance (in cm). The fit coefficients a through k vary with compressed breast thickness, and are given in Table 2. The ability of these equations to predict the RGD(α) values for the tungsten target x‐ray spectra was analyzed to verify if new fit coefficients were needed. This analysis involved computing the mean and maximum absolute differences between the actual computed RGD(α) values for all x‐ray spectra and breast chest‐wall to nipple distance, thickness and composition and the RGD(α) values predicted by the fit equations using the appropriate fit coefficients.

**Table 2:** Coefficients for fit equations (1) and (2) for computation of RGD(α) from Sechopoulos et al.^(10)^

*T(cm)*	*a*	*b*	*c*	*d*	*e*	*f*	*g*	*h*	*i*	*j*	*k*
MLOView											
2	1.0349358E+00	−1.6529060E−02	1.0077362E‐02	6.2692215E‐02	5.8595159E‐02	5.7527400E‐05	−1.7252000E−04	−1.0608000E−03	−9.3921000E−04	8.1035200E‐04	−5.6465000E−04
3	9.8285238E‐01	−2.2306940E−02	1.0055001E‐02	8.4386751E‐02	8.7038980E‐02	8.2108600E‐05	−2.3248000E−04	−1.7703800E−03	−1.8553700E−03	1.1006090E‐03	−5.6204000E−04
4	1.0224779E+00	−2.6531450E−02	1.2824346E‐02	1.1002425E‐01	1.0678169E‐01	1.4712400E‐04	−2.4763000E−04	−2.6128900E−03	−2.4943400E−03	1.1814080E‐03	−8.1818000E−04
5	1.0183296E+00	−2.8581550E−02	1.2678092E‐02	9.5063795E‐02	9.2354960E‐02	1.8802800E‐04	−2.3896000E−04	−2.0215100E−03	−1.9115000E−03	1.1423940E‐03	−9.3090000E−04
6	9.9363885E‐01	−2.8537490E−02	1.4971051E‐02	8.6364355E‐02	8.7895273E‐02	2.4280300E‐04	−2.1764000E−04	−1.8136000E−03	−1.8608200E−03	9.0414300E‐04	−1.2530800E−03
7	9.6518850E‐01	−2.4551700E−02	1.1157589E‐02	1.7101855E‐02	2.3765749E‐02	2.2324400E‐04	−1.8127000E−04	8.2918200E‐04	5.7414100E‐04	6.1190600E‐04	−1.1252000E−03
8	9.472873 5E‐01	−2.0484000E−02	7.9161710E‐03	−3.9651870E−02	−3.0315450E−02	1.9929900E‐04	−1.4532000E−04	2.9771460E‐03	2.6223800E‐03	3.6897700E‐04	−9.8338000E−04
CCView											
2	9.7671287E‐01	−2.8288000E−04	7.0501240E‐03	−1.2163000E−04	−6.5277000E−04	−1.0460000E−05	−3.5244000E−07	1.8795400E‐05	2.2485200E‐06	6.4138300E‐08	
3	9.8639604E‐01	3.8479100E‐04	3.6117490E‐03	−1.4252000E−04	−2.6970000E−04	−1.1892000E−04	−2.0850000E−07	5.7938800E‐06	70957900E‐06	−1.2272000E−06	
4	9.8544839E‐01	9.0665300E‐04	3.4765500E‐03	−1.4728000E−04	−2.3755000E−04	−2.2604000E−04	3.2855500E‐07	4.5156000E‐06	1.2224500E‐05	−3.3923000E−06	
5	9.8793549E‐01	1.8384160E‐03	2.5673120E‐03	−1.3668000E−04	−1.3390000E−04	−3.7845000E−04	2.8577900E‐07	1.1046500E‐06	1.7651900E‐05	4.7866000E‐06	
6	9.5957426E‐01	2.7563230E‐03	1.1131519E‐02	−1.2193000E−04	−9.1140000E−04	−5.1403000E−04	2.8172900E‐07	2.2933100E‐05	2.2262800E‐05	−6.5283000E−06	
7	9.2121146E‐01	3.8140860E‐03	2.1370915E‐02	−7.1292000E−05	−1.7795600E−03	−72343000E−04	−5.3147000E−07	4.6601600E‐05	2.9601500E‐05	−8.4001000E−06	
8	8.7176151E‐01	4.5144410E‐03	3.5520414E‐02	−5.5722000E−05	−3.0308200E−03	−8.4009000E−04	−2.3385000E−07	8.1239700E‐05	3.4711900E‐05	−1.1059000E−05	

RGD(α)=Relative Glandular Dose for projection angle α, MLO=Medio‐Lateral Oblique, CC=Cranio‐Caudal, T=Compressed Breast Thickness

## III. RESULTS

### A. Normalized Glandular Dose at Zero Degree Projection Angle

The resulting $D_{g}N_{0}$ are listed in Table 3. Due to their slow variation with chest wall to nipple distance, the results presented are the mean $D_{g}N_{0}$ for all CND studied. This averaging introduces an error not greater than 6.5% for the MLO view (except for the smallest breast, for which the mean $D_{g}N_{0}$ is up to 14% higher than the actual $D_{g}N_{0}$) and not greater than 3% for the CC view (except for the largest breast for which the mean $D_{g}N_{0}$ is up to 7% higher than the actual $D_{g}N_{0}$). As expected, the $D_{g}N_{0}$ values show the same tendencies as those found for molybdenum and rhodium target acquisitions:^(10)^ increasing $D_{g}N_{0}$ with increasing x‐ray spectrum energy and with decreasing compressed breast thickness and glandular fraction. When comparing $D_{g}N_{0}$ values from tungsten target x‐ray spectra with those published for molybdenum and rhodium target x‐ray spectra with similar HVL after the breast compression plate, the tungsten spectra were found to result in considerably higher $D_{g}N_{0}$, with an increase of up to 18%.^(10)^

**Table 3:** Normalized glandular dose per unit exposure at the intersection of the central ray and the breast support plate (see ^(10)^) for zero degree projection angle, $D_{g}N_{0}$ ($\text{mGy}/2.58\times 10^{-4}C/\text{Kg}$).

*Thickness (cm)*	*Spectrum (Target/Filter/kVp)*	*MLO View Glandularity (%)*					*CC View Glandularity (%)*				
		*1*	*25*	*50*	*75*	*100*	*1*	*25*	*50*	*75*	*100*
2	**W/Al 25**	2.63	2.48	2.34	2.21	2.08	2.91	2.75	2.59	2.44	2.30
	**W/Al 27**	2.92	2.77	2.62	2.48	2.35	3.22	3.05	2.89	2.74	2.59
	**W/Al 29**	3.17	3.02	2.87	2.73	2.59	3.49	3.32	3.16	3.00	2.85
	**W/Al 31**	3.41	3.25	3.10	2.95	2.82	3.74	3.57	3.40	3.25	3.10
	**W/Al 33**	3.62	3.47	3.31	3.17	3.03	3.97	3.80	3.63	3.47	3.32
	**W/Al 35**	3.82	3.66	3.51	3.36	3.22	4.18	4.01	3.84	3.68	3.53
	**W/Rh 25**	3.66	3.47	3.29	3.12	2.96	4.03	3.83	3.63	3.44	3.26
	**W/Rh 27**	3.84	3.66	3.47	3.30	3.13	4.23	4.03	3.82	3.63	3.45
	**W/Rh 29**	3.98	3.79	3.60	3.42	3.25	4.37	4.17	3.96	3.77	3.58
	**W/Rh 31**	4.09	3.90	3.71	3.53	3.36	4.49	4.29	4.08	3.89	3.70
	**W/Rh** 33	4.20	4.01	3.82	3.64	3.47	4.61	4.40	4.19	4.00	3.81
	**W/Rh 35**	4.30	4.11	3.92	3.74	3.57	4.72	4.52	4.31	4.11	3.93
3	**W/Al 25**	2.11	1.94	1.78	1.64	1.51	2.33	2.14	1.97	1.81	1.67
	**W/Al 27**	2.39	2.21	2.04	1.89	1.75	2.63	2.43	2.25	2.08	1.93
	W/Al 29	2.64	2.45	2.28	2.12	1.97	2.90	2.70	2.51	2.33	2.17
	W/Al 31	2.87	2.68	2.50	2.33	2.18	3.16	2.95	2.75	2.56	2.40
	W/Al 33	3.09	2.89	2.71	2.54	2.38	3.39	3.18	2.97	2.79	2.61
	W/Al 35	3.29	3.09	2.90	2.73	2.57	3.61	3.39	3.19	2.99	2.82
	W/Rh 25	3.00	2.78	2.56	2.37	2.19	3.31	3.06	2.83	2.61	2.42
	W/Rh 27	3.18	2.95	2.73	2.53	2.35	3.51	3.25	3.01	2.79	2.59
	W/Rh 29	3.31	3.08	2.85	2.65	2.46	3.65	3.39	3.14	2.92	2.71
	W/Rh 31	3.42	3.19	2.96	2.75	2.56	3.77	3.51	3.26	3.03	2.82
	W/Rh 33	3.53	3.29	3.06	2.85	2.66	3.89	3.62	3.37	3.14	2.93
	W/Rh 35	3.64	3.40	3.17	2.96	2.76	4.01	3.74	3.49	3.25	3.04
4	W/Al 25	1.74	1.57	1.42	1.28	1.17	1.93	1.74	1.57	1.42	1.29
	W/Al 27	1.99	1.81	1.65	1.50	1.37	2.21	2.01	1.82	1.66	1.52
	W/Al 29	2.23	2.04	1.86	1.71	1.57	2.47	2.26	2.06	1.89	1.73
	W/Al 31	2.46	2.26	2.07	1.91	1.76	2.72	2.50	2.29	2.11	1.94
	W/Al 33	2.67	2.46	2.27	2.10	1.94	2.95	2.72	2.51	2.31	2.14
	W/Al 35	2.87	2.66	2.46	2.28	2.12	3.17	2.93	2.71	2.51	2.33
	W/Rh 25	2.50	2.27	2.06	1.87	1.71	2.78	2.52	2.29	2.08	1.89
	W/Rh 27	2.67	2.43	2.21	2.01	1.84	2.96	2.70	2.45	2.23	2.03
	W/Rh 29	2.79	2.55	2.32	2.12	1.94	3.10	2.83	2.57	2.35	2.14
	W/Rh 31	2.90	2.65	2.42	2.21	2.03	3.22	2.94	2.68	2.45	2.24
	W/Rh 33	3.01	2.75	2.52	2.31	2.12	3.33	3.05	2.79	2.55	2.34
	W/Rh 35	3.12	2.86	2.62	2.41	2.21	3.45	3.17	2.90	2.66	2.45
5	W/Al 25	1.47	1.31	1.17	1.05	0.95	1.63	1.45	1.29	1.16	1.04
	W/Al 27	1.71	1.53	1.38	1.24	1.13	1.89	1.70	1.52	1.37	1.24
	W/Al 29	1.93	1.74	1.58	1.43	1.30	2.14	1.93	1.74	1.58	1.43
	W/Al 31	2.14	1.95	1.77	1.61	1.47	2.37	2.15	1.95	1.78	1.62
	W/Al 33	2.35	2.14	1.95	1.79	1.64	2.60	2.37	2.16	1.97	1.80
	W/Al 35	2.54	2.32	2.13	1.96	1.80	2.81	2.57	2.35	2.16	1.98
	W/Rh 25	2.14	1.91	1.71	1.54	1.39	2.37	2.12	1.90	1.70	1.53
	W/Rh 27	2.29	2.06	1.85	1.66	1.50	2.55	2.28	2.05	1.84	1.66
	W/Rh 29	2.41	2.16	1.94	1.76	1.59	2.67	2.40	2.16	1.94	1.76
	W/Rh 31	2.51	2.26	2.03	1.84	1.67	2.78	2.51	2.26	2.04	1.84
	W/Rh 33	2.61	2.35	2.13	1.93	1.75	2.89	2.61	2.36	2.13	1.94
	W/Rh 35	2.71	2.46	2.22	2.02	1.84	3.01	2.73	2.47	2.24	2.04
6	W/Al 25	1.27	1.13	1.00	0.89	0.79	1.41	1.24	1.10	0.98	0.87
	W/Al 27	1.49	1.33	1.18	1.06	0.95	1.65	1.47	1.31	1.17	1.05
	W/Al 29	1.70	1.52	1.36	1.23	1.11	1.88	1.68	1.50	1.35	1.22
	W/Al 31	1.90	1.71	1.54	1.39	1.26	2.10	1.89	1.70	1.53	1.39
	W/Al 33	2.09	1.89	1.71	1.55	1.41	2.32	2.09	1.89	1.71	1.56
	W/Al 35	2.27	2.07	1.87	1.71	1.56	2.52	2.28	2.07	1.89	1.72
	W/Rh 25	1.86	1.65	1.46	1.30	1.17	2.06	1.83	1.62	1.44	1.29
	W/Rh 27	2.00	1.78	1.58	1.41	1.27	2.22	1.97	1.75	1.56	1.40
	W/Rh 29	2.11	1.87	1.67	1.50	1.35	2.34	2.08	1.85	1.65	1.48
	W/Rh 31	2.20	1.96	1.75	1.57	1.42	2.44	2.18	1.94	1.74	1.56
	W/Rh 33	2.29	2.05	1.84	1.65	1.49	2.55	2.28	2.03	1.83	1.65
	W/Rh 35	2.40	2.15	1.93	1.74	1.57	2.66	2.38	2.14	1.92	1.74
7	W/Al 25	1.13	0.99	0.87	0.77	0.69	1.24	1.09	0.95	0.84	0.75
	W/Al 27	1.33	1.17	1.04	0.92	0.83	1.47	1.29	1.14	1.02	0.91
	W/Al 29	1.52	1.35	1.20	1.08	0.97	1.68	1.49	1.32	1.18	1.06
	W/Al 31	1.71	1.52	1.36	1.23	1.11	1.89	1.68	1.50	1.35	1.22
	W/Al 33	1.89	1.69	1.52	1.37	1.24	2.09	1.87	1.68	1.52	1.37
	W/Al 35	2.06	1.86	1.68	1.52	1.38	2.28	2.06	1.85	1.68	1.52
	W/Rh 25	1.65	1.45	1.28	1.13	1.01	1.82	1.60	1.41	1.25	1.11
	W/Rh 27	1.78	1.57	1.38	1.23	1.10	1.97	1.73	1.53	1.35	1.21
	W/Rh 29	1.87	1.66	1.47	1.31	1.17	2.07	1.83	1.62	1.44	1.28
	W/Rh 31	1.96	1.74	1.54	1.37	1.23	2.17	1.92	1.70	1.51	1.35
	W/Rh 33	2.05	1.82	1.62	1.45	1.30	2.27	2.01	1.79	1.59	1.43
	W/Rh 35	2.15	1.91	1.71	1.53	1.38	2.38	2.12	1.88	1.69	1.52
8	W/Al 25	1.00	0.88	0.77	0.68	0.60	1.11	0.97	0.84	0.74	0.66
	W/Al 27	1.19	1.05	0.92	0.82	0.73	1.32	1.15	1.02	0.90	0.80
	W/Al 29	1.37	1.21	1.07	0.96	0.86	1.52	1.34	1.18	1.05	0.94
	W/Al 31	1.54	1.37	1.22	1.09	0.98	1.71	1.52	1.35	1.21	1.08
	W/Al 33	1.71	1.53	1.37	1.23	1.11	1.90	1.70	1.51	1.36	1.23
	W/Al 35	1.88	1.69	1.51	1.37	1.24	2.09	1.87	1.68	1.51	1.37
	W/Rh 25	1.47	1.29	1.13	1.00	0.89	1.63	1.42	1.25	1.10	0.98
	W/Rh 27	1.59	1.39	1.23	1.09	0.97	1.76	1.54	1.35	1.20	1.06
	W/Rh 29	1.68	1.48	1.30	1.15	1.03	1.86	1.63	1.44	1.27	1.13
	W/Rh 31	1.76	1.55	1.37	1.22	1.09	1.95	1.72	1.51	1.34	1.20
	W/Rh 33	1.85	1.63	1.44	1.28	1.15	2.05	1.81	1.59	1.42	1.27
	W/Rh 35	1.94	1.72	1.52	1.36	1.22	2.15	1.90	1.69	1.50	1.35

CC=Cranio‐Caudal, MLO=Medio‐Lateral Oblique, CND=chestwall to nipple distance. 2.58×10−4C/kg=1 Roentgen=8.76 mGy air kerma

### B. Relative Glandular Dose Coefficients for the Non‐Zero Projection Angles

**Figure 1:**
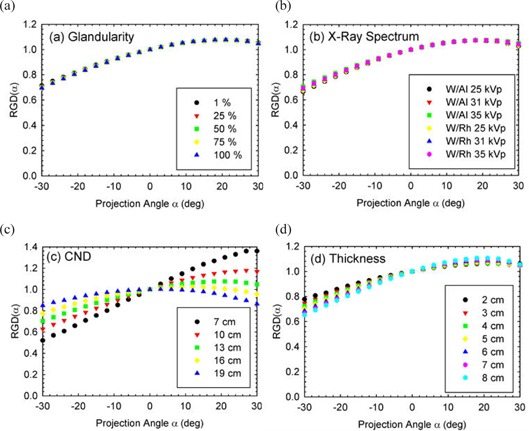
RGD(α) in the MLO view. Graphs of variation of RGD(α) in the MLO view with (a) breast glandular fraction, (b) x‐ray spectrum, (c) chest wall to nipple distance (note the different y‐axis scale), and (d) compressed breast thickness. Positive tomosynthesis angles occur when the x‐ray tube is closer to the cranial side of the patient. The parameters, unless varied, are: 50% breast glandular fraction, W/Al 33 kVp spectrum, CND=10 cm, and breast thickness=5 cm.

**Figure 2:**
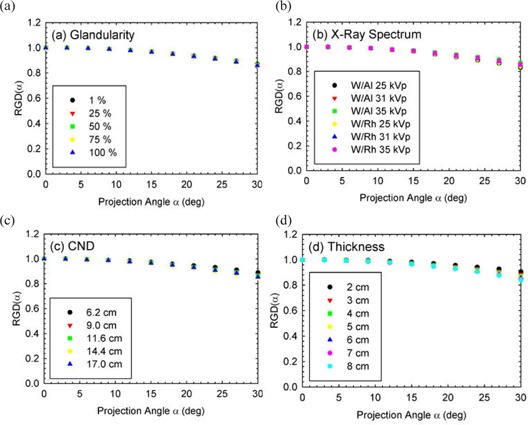
RGD(α) in the CC view. Graphs of variation of RGD(α) in the CC view with (a) breast glandular fraction, (b) x‐ray spectrum, (c) chest wall to nipple distance, and (d) compressed breast thickness. Due to geometrical symmetry, only the positive tomosynthesis angles were simulated for the CC view breast. The parameters, unless varied, are: 50% breast glandular fraction, W/Al 33 kVp spectrum, CND=11.6 cm, and breast thickness=5cm.

Figures 1 and 2 depict the variation of RGD(α) for different breast glandular fraction, x‐ray spectrum, chest wall to nipple distance, and compressed breast thickness, for the MLO and CC view, respectively. As expected, similar influence of the four parameters on the RGD(α) values as those previously reported for the molybdenum and rhodium target spectra was found.^(10)^ In addition, the results of the quantitative analysis, in the form of the coefficients of variation and deviations from the mean when varying these parameters individually are shown in Table 4. These again show very similar results to those previously published for the different x‐ray spectra.^(10)^

**Table 4:** Variation of RGD(α) with the studied parameters.

*View*	*Parameter*	*Coefficient of Variation*	*Deviation from Mean*
		*Mean*	*Maximum*	*Mean*	*Maximum*
MLO	Glandularity	0.27%	1.43%	0.37%	1.91%
	X‐ray Spectrum	0.48%	2.01%	0.92%	3.76%
	Thickness	2.72%	12.48%	4.16%	19.86%
	CND	9.89%	24.85%	13.99%	36.27%
CC	Glandularity	0.19%	0.86%	0.26%	1.13%
	X‐ray Spectrum	0.39%	1.31%	0.75%	2.60%
	Thickness	1.01%	4.75%	1.64%	7.37%
	CND	0.87%	6.42%	1.28%	9.38%

RGD(α)=Relative Glandular Dose for projection angle a, CC=Cranio‐Caudal, MLO=Medio‐Lateral Oblique, CND=Chest wall to nipple distance.

Table 5 shows the mean and maximum absolute differences found between the actual computed RGD(α) values and those predicted by Equations (1) and (2). The data shows that the surface fits reported in the previous study to predict RGD(α) are appropriate for tungsten target x‐ray spectra.

**Table 5:** Accuracy of prediction of RGD(α) by fitted equations.

*View*	*Study*	*Maximum Difference*	*Mean Difference*
MLO	Previous Study (Mo and Rh targets)^(10)^	8.37%	0.88%
	Current Study (W targets)	8.86%	0.96%
CC	Previous Study (Mo and Rh targets)^(10)^	4.08%	0.58%
	Current Study (W targets)	5.23%	0.74%

RGD(α)=Relative Glandular Dose for projection anglea, CC=Cranio‐Caudal, MLO=Medio‐Lateral Oblique.

## IV. DISCUSSION

X‐ray spectra generated by x‐ray tubes with tungsten targets have a very different x‐ray distribution from those generated using molybdenum or rhodium targets. Therefore, it was expected that the dosimetric characteristics of tomosynthesis imaging with tungsten targets would be different from those resulting from imaging with targets of these other materials, even with x‐ray spectra with similar or equal first half value layers. The normalized glandular dose values for the zero projection angle ($D_{g}N_{0}$) computed here for x‐ray spectra with tungsten targets reflect these differences. Not only are the $D_{g}N_{0}$ values for x‐ray spectra with corresponding tube voltage settings in general higher, which is expected due to the higher first half value layers, but, due to the difference in shape, x‐ray spectra with matched HVL result in normalized glandular dose values for the zero projection angle up to 18% higher. These differences must be taken into account when performing comparison or optimization studies between tomosynthesis imaging systems with different target materials.

As opposed to the $D_{g}N_{0}$, the RGD(α) values were found not to vary considerably from those found in the previous study. This was expected given the very low sensitivity of RGD(α) to the x‐ray spectrum used.^(10)^ Therefore, it was found that although the zero degree projection data specific for the tungsten target x‐ray spectra needs to be used when appropriate, the surface fit equations, with their corresponding fit coefficients (published in the previous study^(10)^ and re‐published here) are applicable for calculations with any of the three target materials.

It must be noted that although the normalized glandular dose of tungsten target spectra is in general higher than that of spectra with other targets both for x‐ray spectra with equal tube voltage or equal first half value layer, rhodium‐filtered tungsten target x‐ray spectra result in much lower exposure values for a given tube current setting. Therefore the actual glandular dose from an imaging study with rhodium‐filtered tungsten x‐ray spectra is considerably lower when compared to an imaging study performed with other materials but equivalent tube current. Although this is beneficial, the impact that the selection of this target/filter combination has on image contrast must also be taken into account.

Our study involved the use of previously generated monochromatic dosimetry data obtained using Monte Carlo simulations. Therefore, the results published here share the same limitations of most Monte Carlo breast dosimetry studies. The most important of these is the definition of the composition of the breast as a homogeneous mixture of glandular and adipose tissue. It has been established that if the breast composition were defined as a heterogeneous mixture of these two tissue types, the relative position of the glandular tissue in the breast can introduce an important variation in glandular dose.^(18)^ However, this study, in a similar fashion as the other Monte Carlo breast dosimetry studies, aims to characterize the dose involved in a breast imaging technique in general, allowing for comparison with other imaging techniques, not for specific dose estimations for specific patients.

## V. CONCLUSIONS

To complete the availability of the data necessary to perform dosimetry calculations of tomosynthesis imaging of the breast, we used previously obtained monochromatic normalized glandular dose values obtained from Monte Carlo simulations of tomosynthesis imaging to compute the normalized glandular dose for relevant x‐ray spectra generated by tungsten targets. This completes the previously reported dosimetry model, allowing for the calculation of glandular dose for any kind of tomosynthesis acquisition protocol, including new advanced protocols that may use variable tube current and/or voltage settings, which are already being investigated.^(26)^
